# Identification of circulatory biomarkers from clinical trial samples

**DOI:** 10.1186/2051-1426-3-S2-P104

**Published:** 2015-11-04

**Authors:** Laura Rosa Brunet, Katherine Bodman-Smith, Mark Bodman-Smith, Zain Kassam, Angus Dalgleish, Sam LaBrie, Thorsten Hagemann

**Affiliations:** 1 Immodulon Therapeutics Ltd, Uxbridge, UK; 2 University of Surrey, Guildford, UK; 3 St' George's University of London, London, UK; 4 Myriad RBM, Ausin, TX, USA

## Background

Despite significant advances in genomics, proteomics and metabolomics, the identification and validation of circulatory immunological biomarkers in cancer patients, which could be useful to monitor disease progression and response to immunotherapeutic treatments, remains as elusive as ever. Circulatory biomarkers, being easily accessible, may provide crucial guidance and be incorporated in routine point of care practices. Immunological biomarkers could aid clinical decision making regarding initiation, cessation, boost or change of treatment and assessment of therapeutic responses. In light of the increasing number of immunotherapeutic options, this is an area receiving renewed attention.

## Methods

We have investigated two different approaches to identify circulating immunological mediators in clinical samples. We have measured immunological mediators by ELISA or Multiplex assays in either the sera or in the supernatants of TruCulture^®^ samples from patients with pancreatic (NCT01303172) and colorectal cancer (NCT01539824) recruited to clinical trials investigating the use of IMM-101, an immunotherapeutic agent. IMM-101, a suspension of heat-killed whole cell *Mycobacterium obuense* (NCTC13365) for intra-dermal injection, is a systemic immunomodulator which has effects on innate and adaptive immune responses and which may have application across a variety of tumor types. The TruCulture^®^ system assesses immune cell activity within a single self-contained sterile tube containing immunological stimulants, such as SEB and anti-CD28, and requiring an incubation step of 24hrs in a dry-heat block.

## Results

Nursing staff reported that the tubes were simple to manage, the protocol easy to perform at point of care and sample acquisition was well integrated within the clinical trial protocol. Supernatants were stored until ready for analysis. Data revealed that this technique offers a way of reliably measuring immunological responses by patients before and after treatment. Compared to multiplex analysis of sera samples, which detected only low levels of a very limited range of cytokines, data show that this system allows for detection of a full range of cytokines (e.g. IFN-γ, IL-2, IL-4, IL-7) and that it is a promising technique to monitor patients' immunological responses. Sera analysis by ELISA and Multiplex assay did reveal the presence of only low measurable levels of cytokines, while levels of chemokines (e.g. MCP-1, MIP-1α and MIP-1β) were consistently detected. We are currently analysing samples for inflammatory mediators.

## Conclusions

Preliminary data indicate that novel inflammatory markers such as Apolipoprotein E, may be used to monitor treatment response. It is likely, however, that patterns of markers may prove potentially more useful than individual markers.

